# Structural Competency in Mental Health and Medicine: A Case-Based Approach to Treating the Social Determinants of Health

**DOI:** 10.5195/jmla.2021.1212

**Published:** 2021-04-01

**Authors:** Gregory Laynor

**Affiliations:** 1 gregory.laynor@jefferson.edu, Scott Memorial Library, Thomas Jefferson University, Philadelphia, PA

There has been increasing awareness in the health professions of the need for “cultural competency,” such as attention to the impact of unconscious bias in the interactions of health care providers and patients. There has also been an increasing awareness of the limitations of cultural competency as a framework for addressing inequities in healthcare.

In 2014, Helena Hansen and Jonathan Metzl coined the term “structural competency” [1]. Hansen is a psychiatrist who is also trained as a medical anthropologist; Metzl is a psychiatrist who is also trained as a cultural historian. Hansen and Metzl's framework of structural competency builds upon expertise from the social sciences and humanities as well as the tradition of social medicine, which sees health as based in social and economic conditions. Whereas cultural competency involves the awareness of bias at the level of the individual, structural competency expands this awareness to the economic and social structures underlying health.

Structural competency might at first sound overly abstract or too ambitious. The 17 case studies collected in Hansen and Metzl's *Structural Competency in Mental Health and Medicine: A Case-Based Approach to Treating the Social Determinants of Health* show how clinicians, medical educators, and interprofessional collaborators have put structural competency into practice in the real world. Each case study identifies a problem, a path to addressing the problem, and lessons learned. Cases include curriculum development, integrations of clinical care with community advocacy, and collaborations between clinicians and organizations such as healthcare labor unions and immigrant rights groups.

A highlight of the book is “Teaching and Testing Structural Competency in Pre-health Undergraduate Classrooms,” a case study on the innovative undergraduate degree in medicine, health, and society that Metzl and his colleagues have developed at Vanderbilt University. An alternative to a conventional pre-med or pre-health professions curriculum, the program starts developing structural competency at the undergraduate level by integrating foundational biomedical science courses with courses on the social and economic conditions of health along with community health site visits and service learning. Another case, “Is Poverty Making Me Sick? An Example of the Impact of Medical-Legal Partnership on Keeping Children Healthy,” describes a successful collaboration between Cincinnati Children's Hospital and the Legal Aid Society of Greater Cincinnati. Pediatrics residents collaborate with legal aid advocates to apply legal expertise to the structural drivers of their patients’ health, such as taking landlords to court to remove mold and negotiating with state bureaucrats to get food assistance. Overall, the cases in the book show multiple ways that collaborations across professions, knowledges, institutions, and communities can help achieve structural competency.

A helpful starting point for learning more about the need for structural competency is the book's foreword, written by Dorothy Roberts, author of *Fatal Invention: How Science, Politics, and Big Business Re-create Race in the Twenty-first Century* and *Killing the Black Body: Race, Reproduction, and the Meaning of Liberty*. The book also includes a very useful bibliography, with further resources on developing structural competency beyond cultural competency, key articles on the social determinants of health, additional examples of implementing structural competency, and related films and websites. As the first collection of case studies on structural competency, the book models an approach to healthcare that has become even more relevant during a pandemic, in which the deadly effects of structural racism are hard to ignore [2].

The book would be a worthwhile addition to any collection on cultural competency; diversity, equity, and inclusion; public health; social determinants of health; or health professions education. Hansen and Metzl's *Structural Competency in Mental Health and Medicine: A Case-Based Approach to Treating the Social Determinants of Health* will, I hope, inspire further development and implementation of structural competency perhaps involving collaborations with health sciences libraries and librarians.
